# Leaf Economic and Hydraulic Traits Signal Disparate Climate Adaptation Patterns in Two Co-Occurring Woodland Eucalypts

**DOI:** 10.3390/plants11141846

**Published:** 2022-07-14

**Authors:** Suzanne M. Prober, Brad M. Potts, Peter A. Harrison, Georg Wiehl, Tanya G. Bailey, João Costa e Silva, Meridy R. Price, Jane Speijers, Dorothy A. Steane, René E. Vaillancourt

**Affiliations:** 1 CSIRO Land and Water, Private Bag 5, Wembley, WA 6913, Australia; georg.wiehl@csiro.au (G.W.); dorothy.steane@utas.edu.au (D.A.S.); 2 School of Natural Sciences, University of Tasmania, Private Bag 55, Hobart, TAS 7001, Australia; b.m.potts@utas.edu.au (B.M.P.); p.a.harrison@utas.edu.au (P.A.H.); tanya.bailey@utas.edu.au (T.G.B.); meridy.price@utas.edu.au (M.R.P.); rene.vaillancourt@utas.edu.au (R.E.V.); 3 ARC Training Centre for Forest Value, University of Tasmania, Private Bag 55, Hobart, TAS 7001, Australia; 4 Centro de Estudos Florestais, Instituto Superior de Agronomia, Universidade de Lisboa, Tapada da Ajuda, 1349-017 Lisboa, Portugal; jces@isa.ulisboa.pt; 5 McDonald Speijers, Mining, Geological and Statistical Consultants, 11a Swanbourne Str., Fremantle, WA 6160, Australia; jane@mcsp.com.au

**Keywords:** assisted migration, climate adaptation, *Eucalyptus*, hydraulic traits, leaf traits, provenancing strategies, seed-sourcing, parallel evolution

## Abstract

With climate change impacting trees worldwide, enhancing adaptation capacity has become an important goal of provenance translocation strategies for forestry, ecological renovation, and biodiversity conservation. Given that not every species can be studied in detail, it is important to understand the extent to which climate adaptation patterns can be generalised across species, in terms of the selective agents and traits involved. We here compare patterns of genetic-based population (co)variation in leaf economic and hydraulic traits, climate–trait associations, and genomic differentiation of two widespread tree species (*Eucalyptus pauciflora* and *E. ovata*). We studied 2-year-old trees growing in a common-garden trial established with progeny from populations of both species, pair-sampled from 22 localities across their overlapping native distribution in Tasmania, Australia. Despite originating from the same climatic gradients, the species differed in their levels of population variance and trait covariance, patterns of population variation within each species were uncorrelated, and the species had different climate–trait associations. Further, the pattern of genomic differentiation among populations was uncorrelated between species, and population differentiation in leaf traits was mostly uncorrelated with genomic differentiation. We discuss hypotheses to explain this decoupling of patterns and propose that the choice of seed provenances for climate-based plantings needs to account for multiple dimensions of climate change unless species-specific information is available.

## 1. Introduction

Anthropogenic acceleration of global climate change raises key questions regarding the capacity of native plant species to withstand or adapt to such change [1,2]. Tree species are of particular concern due to their longevity, often poor dispersal capacity, role as foundation species in many terrestrial ecosystems, as well as their importance in ecological renovation (*sensu* [3]) and global carbon sequestration [1,4]. Developing an understanding of adaptive capacity of tree populations can guide intervention strategies to facilitate their adaptation and resilience in the face of climate change [5,6,7,8,9]. An increasing body of research has begun to characterise adaptive variation within tree species related to climate [5,7,10,11,12]. Such studies aim to develop a predictive understanding of climate adaptation, and ultimately heuristic guidelines that managers can use to guide seed-source (provenance) choices for forestry and ecological renovation. In particular, the choice of species and provenances may be guided by the characteristics of the home-site climate and covariation of functional traits with climate. Developing such heuristics requires empirical data on climate/trait associations across suites of species with differing characteristics.

The extent to which the patterns of adaptive variation in functional traits are predictable from niche differences is a key question in evolutionary biology, and underlies studies of parallel/convergent evolution at the species and population levels [13,14,15,16]. Repeated genetic-based patterns of trait variation in independent populations occupying similar environments is strong evidence for the influence of natural selection, as the same trait-environment associations are unlikely to repeatedly arise through neutral processes such as drift and mutation [13,17]. However, empirical studies addressing this issue within species often report a continuum in ‘parallel and nonparallel’ (or ‘shared and unique’) patterns of trait divergence [16,18,19]. Further, the specific functional traits exhibiting population divergence may not be as anticipated, with reported cases of both adaptive differentiation and evolutionary stasis reported [4,20].

Differences in the patterns of (co)variance among traits will reflect different patterns and processes of trait integration (e.g., developmental, functional, genetic, and evolutionary integration) that may occur at multiple levels (species, populations within species, or individuals within populations) [21,22]. In addition, environmental gradients may confound different selective forces (e.g., aridity gradients can involve covarying temperature and precipitation [23]). This can lead to ‘hidden’ selection [13,19,24], further complicating the predictability of the trajectory of phenotypic evolution in response to climate change. Nevertheless, there are many empirical studies revealing broad patterns of parallel/convergent evolution and multitrait coordination within animal (e.g., [19,25]) and plant (e.g., [11,26]) species. Adaptive syndromes are often hypothesised [4,27,28,29], which combined with the empirical findings provides impetus for the goal of predicting adaptive phenotypes for future climates [4,26]. 

In addition to life-history traits [30], a predictive understanding of climate adaptation also requires consideration of different groups of functional traits. In plants, multiple traits are known to affect performance and stress adaptation, including leaf economic and hydraulic traits. Leaf economic traits have been shown to affect resource allocation strategies with various trait combinations hypothesised to reflect different returns on the investment in nutrients and dry mass in leaves, which includes a continuum from species with competitive (‘fast species’) to conservative (‘slow species’) growth strategies [27,31]. Leaves of ‘slow species’ are often more sclerotic and are expected to have longer lifespans and higher survival in the face of abiotic and biotic stress [29,32]. The leaf hydraulic traits such as stomatal and vein density affect leaf gas exchange (e.g., CO_2_ uptake) and water transfer [33,34], with higher densities promoting faster growth. 

Emerging empirical evidence shows little support for coordination between leaf economic and hydraulic traits [33,35]. Rather, accumulating evidence suggests different traits provide different adaptation opportunities, resulting in a wide diversity of adaptation options through differential combinations of traits [36,37]. However, functional trait (co)variation and trait-climate associations may change with scale [32,38]. Most theoretical and empirical studies involving the leaf economic and hydraulic traits have focused on higher-level community or species level associations (e.g., [29,31,39]). In trees, attention is increasingly shifting to associations at the intra-specific level [40,41,42], particularly the genetic-based differences and associations between populations [35,43] that provide insights into micro-evolutionary responses to climate variation [44]. 

The present study used a common-garden experiment to compare variation in growth performance, leaf economic, and leaf hydraulic traits from populations of two widespread eucalypt species, *Eucalyptus pauciflora* Sieber ex Spreng. and *E. ovata* Labill., native to the island of Tasmania, Australia. As we were interested in a direct comparison of the two species where they experience the same climate gradient(s), we focused our study on a set of 22 paired populations from the overlapping geographic range of these species. We specifically aimed to compare species in terms of their patterns of: (i) genetic-based population variation and covariation in leaf traits; (ii) climate-trait associations of leaf traits; and (iii) genomic differentiation among populations. We hypothesised that:

(a) the two species will share similar patterns of trait (co)variation and trait–climate relationships owing to their co-occurrence in a common climate landscape;
(b) leaf economic and leaf hydraulic traits will have different axes of variation within species, consistent with emerging patterns of variation between species [33,35];
(c) trends in leaf economic and hydraulic traits will reflect expectations for adaptations to climate.

## 2. Results

### 2.1. Climate Landscape

Our samples of the 2 species did not differ significantly (*p* > 0.05) in altitude or in the 10 climatic variables studied (Table S1), and the average correlation among co-located species populations for the 10 climatic variables was 0.97 (range 0.95–1.00). From this we concluded that the sampled populations of both species were from the same (macro)climate landscape and experienced the same climate gradient(s), which was the intent of our paired sampling design.

### 2.2. Species Trait Differences

When the populations sampled were grown in a common garden, the two species exhibited highly significant (*p* < 0.001) differences in growth performance measures, as well as in most leaf economic and hydraulic traits (Table 1). These species differences reflected a marked, genetic-based difference in growth strategies. The greater growth rate (height and stem diameter) and higher leaf SLA of *E. ovata* (mainly due to its thinner leaves) was consistent with a less conservative growth strategy compared with *E. pauciflora*. On average, the leaves of *E. ovata* also had a greater density of stomata and veins (at *p* < 0.001, in both cases) compared to the leaves of *E. pauciflora*. However, the stomata of *E. pauciflora* were larger than those of *E. ovata* (*p* < 0.001), on average, resulting in no statistically significant difference between the species in the total stomata length per unit leaf area (*p* > 0.05).

### 2.3. Population Variation

No statistically significant among-population variance (*p* > 0.05) in performance traits was detected at two years of age within either species in the fitted linear mixed models (but see relationships with climate of origin, below) (Table 2). In contrast, among-population variance was significant at the 5% nominal level for five of eight leaf traits in *E. pauciflora*, and three of eight leaf traits in *E. ovata* (Table 2). While more leaf traits exhibited significant among-population variance in *E. pauciflora* than in *E. ovata*, variance homogeneity tests indicated that differences between species in population variances were only statistically significant for leaf area (*p* < 0.01) and leaf thickness (*p* < 0.05). The compound traits SLA and stomata length per area were the only traits that exhibited significant population variance (*p* < 0.05) in both species.

Even when growing in the same climate landscape and experiencing the same climate gradients, the paired population means of the two species were uncorrelated at the 5% nominal level (Table 2). In particular, even for the traits where population variance was detected to be statistically significant in both species (i.e., SLA and stomata length per area), we found no significant correlations (*p* > 0.05) among the paired population means of the two species.

The greater significance of the genetic-based population variability in leaf traits in *E. pauciflora* than in *E. ovata* was confirmed at the multivariate level (Table 3). Using the set of primary economic (leaf area, thickness, and density) and hydraulic (stomata density, stomata length, and vein density) traits, MANOVA showed marginal nonsignificant generalized variance among populations in *E. ovata* (*p* = 0.081), but this was highly significant in *E. pauciflora* (*p* < 0.001). The species difference was mainly driven by the leaf economic traits, for which the generalized variance among populations was highly significant in *E. pauciflora* (*p* < 0.001) but not significant in *E. ovata* (*p* = 0.333). While the species difference was clearly weaker, the reverse trend was evident for the hydraulic traits where the generalized variance of *E. ovata* populations was marginally significant (*p* = 0.021) but not significant in *E. pauciflora* (*p* = 0.152). Thus, in the same landscape, this finding indicates that suites of primary leaf traits are contributing differently to genetic-based population differentiation in the two species (Table 3), which is also consistent with the results on intra-class correlations estimated for individual traits (Table 2).

### 2.4. Correlations among Traits

Within species, the population means of the three primary leaf economic traits were not statistically significantly correlated at the 5% nominal level (Table 4). In contrast, while most combinations of the three primary hydraulic traits were statistically uncorrelated within species, population-mean vein density and stomata density were positively correlated in *E. ovata* (r = 0.55, *p* < 0.01), signalling some coordination of hydraulic traits with respect to gas exchange and photosynthesis. In both species, there was also a statistically nonsignificant trend at the population level for high stomata density to be associated with low stomata size. 

Population means in the composite traits—SLA and stomata length per unit area— were often highly significantly (*p* < 0.001) correlated with one or other of their component primary traits in a consistent manner in both species (Table 4). For example, increases in population mean SLA were mainly associated with reduced leaf thickness (r < −0.8; *p* < 0.001) and reduced leaf density (r < −0.6; *p* < 0.001), but not significantly correlated with leaf area. This indicates that it is the population differences in leaf density, and particularly in leaf thickness, which underlies the population differences in SLA.

There were only a few significant correlations within species reflecting population-level covariation of an economic trait with a hydraulic trait (Table 4). Several of these correlations were notable, as they involved opposite or different patterns of covariation among population means in the two species. For example, thicker-leaved populations of *E. ovata* tended to have a greater length of stomata per unit area (r = 0.44, *p* < 0.05), meaning more potential for gas exchange but also water loss, whereas the opposite was the case in *E. pauciflora* (r = −0.53, *p* < 0.01), indicating thinner-leaved populations have more potential for gas exchange and water loss. These associations did not transfer to a significant correlation with SLA in *E. ovata* but did in *E. pauciflora* where populations with leaves with higher mean SLA tended to have a greater length of stomata per unit area (r = 0.51, *p* < 0.05), mainly due to their greater stomata density (r = 0.45, *p* < 0.05). Thus, across the same climate landscape, not only do the species differ in their patterns of univariate (Table 2) or multivariate population variability (Table 3), but also in the pattern of covariation among traits within species (Table 4).

### 2.5. Comparisons of Molecular and Quantitative Differentiation among Populations

The significant population differentiation detected in quantitative traits in the two species was also evident in molecular data where low, but significant, genome-wide SNP differentiation (F_ST_) was detected among the populations sampled within both species. While based on different genome-wide SNP sets, the overall F_ST_ among the *E. pauciflora* populations (21 populations; F_ST_ = 0.030, 95% CI 0.026–0.033; based on 3603 DARTseq markers) was significantly higher than that among the *E. ovata* populations (21 populations; F_ST_ = 0.013, 95% CI 0.011–0.015; based on 22,708 Euc72K chip markers), consistent with its greater population differentiation in leaf traits. A slightly lower, but significant overall F_ST_ for *E. pauciflora*, was also obtained using the 10 putative neutral microsatellite loci assayed by Gauli et al. [45] (21 populations; F_ST_ = 0.024, 95% CI 0.017–0.032). These data were from the same *E. pauciflora* trees as assayed for SNPs, and the pairwise F_ST_ values were significantly positively correlated at the population level (Spearman Mantel correlation, rho = 0.55, *p* = 0.003).

The discordant patterns of population variation between species observed in leaf functional traits also extended to the genomic level, where there was no significant association of the patterns of population differentiation in the co-occurring *E. ovata* and *E. pauciflora* populations. The Mantel correlation of the matrices of pairwise SNP-derived F_ST_ values among populations of the two species (linked by their geographically paired populations) was not significantly different from zero (Table 5). The equivalent matrices of generalised distances calculated from various groupings of quantitative traits for each species were similarly uncorrelated between *E. ovata* and *E. pauciflora* (Table 5), consistent with the absence of significant correlations among population means of the species for the leaf traits at the univariate level (Table 2). This independence of the multivariate patterns of pairwise population differentiation in quantitative traits was evident regardless of whether the primary hydraulic, economic, or all traits were compared. Focusing on the population differences for the trait types that were significant in the MANOVA (Table 3), we found no significant correlation between the generalised distance matrix calculated among the populations based on leaf hydraulic traits for *E. ovata* and leaf economic traits for *E. pauciflora*, arguing against the species showing parallel but alternative adaptation strategies. 

The among-population SNP-derived F_ST_ and trait-based generalised distance matrices were also uncorrelated within *E. pauciflora* (Table 5), which signals a role for directional selection in shaping the significant population divergence detected in economic traits. This was similarly the case for the hydraulic traits in *E. ovata*, which were also significantly different among populations (Table 3). However, in *E. ovata* there was a significant Mantel correlation between the pairwise F_ST_ values and the nonsignificant (as suggested by the results of Table 3) generalised distances based on the primary leaf economic traits (Table 5), raising the possibility that neutral population processes have contributed to the observed variation among populations, which was statistically insignificant at the univariate (Table 2) and multivariate (Table 3) levels. However, we argue that differentiation in the leaf economic traits is still likely to reflect a weak underlying adaptive signal, because leaf economic traits in *E. ovata* were substantially and significantly related to climate (see below) despite lack of statistical significance detected for the among-population variation.

### 2.6. Discriminant Analysis and Projections of Home-Site Climate Vectors

**Figure 1:**
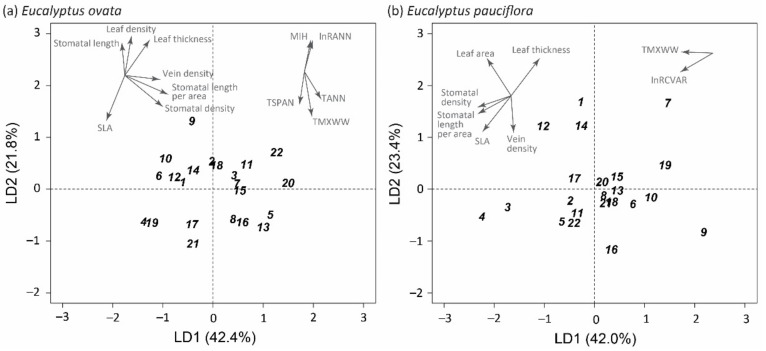
Plots of population centroids in the space defined by the first two axes (LD1 and LD2) derived from separate discriminant analyses of the 22 paired populations of *E. ovata* ((**a**): LD1 42.4%, *p* = 0.081; LD2 21.8%, *p* = 0.690) and *E. pauciflora* ((**b**): LD1 42.0%, *p* < 0.001; LD2 23.4%, *p* = 0.045). The discriminant analyses were based on the six primary leaf economic and hydraulic traits, and the resulting LD axes are independent maximum variance axes. The direction of trait (primary and compound traits) and climate vectors which showed a significant (*p* < 0.05) fit into the two-dimensional discriminant space are indicated (See Table S2). The length of each arrow is proportional to the correlation between the ordination and the trait/climatic variable, and the direction of each arrow is the direction of its most rapid change [46]. The population codes (1-22) correspond to the spatially paired populations as detailed in Table S3.

Discriminant analysis showed different trait-based patterns of variation among populations in *E. ovata* compared with *E. pauciflora* (Figure 1; Procrustean r = 0.210, *p* = 0.658, for the two-dimensional result). In *E. ovata,* population differentiation in leaf traits was marginally nonsignificant (*p* ≤ 0.1) on the first maximum variance axis (LD1 42.4%, *p* = 0.081; LD2 21.8%, *p* = 0.690), whereas in *E. pauciflora* statistically significant differentiation occurred in two independent directions (LD1 42%, *p* < 0.001, and LD2 23.4%, *p* = 0.045) (Figure 1). 

There were similarities and differences between species in the relationships between climate and traits in the discriminant space defined by the leaf traits. In *E. ovata*, the two-dimensional discriminant space was most closely aligned with three temperature vectors, particularly maximum temperature (TMXWW, R^2^ = 0.56, *p* < 0.001), opposed weakly by precipitation (lnRANN, R^2^ = 0.28, *p* = 0.042) and the moisture index (MIH, R^2^ = 0.28, *p* = 0.040) (Figure 1, Table S2). The vectors for stomata density (R^2^ = 0.96, *p* < 0.001) and SLA (R^2^ = 0.92, *p* < 0.001) had the best fit (Table S2).

In *E. pauciflora*, vector fitting similarly revealed an alignment of the discriminant space with maximum temperature (TMXWW, R^2^ = 0.30, *p* = 0.034), but this was weaker and oriented differently compared with the trend in *E. ovata*. Further, precipitation seasonality was significantly associated with population variation in the two-dimensional discriminant space for *E. pauciflora* (lnRCVAR, R^2^ = 0.42, *p* = 0.007), compared with a corresponding statistically nonsignificant result for *E. ovata* (R^2^ = 0.15, *p* = 0.215). For *E. pauciflora*, vectors for specific leaf area (R^2^ = 0.82, *p* < 0.001) and leaf thickness (R^2^ = 0.86, *p* < 0.001) had the best fit to the discriminant space (Table S2).

### 2.7. Univariate Climate-Trait Associations

Further evidence that patterns of leaf trait variation in the two species are aligned with different facets of climate variation in the common landscape comes from the modelling of climate-trait associations using multiple linear regression analyses (Table 6). In contrast to analyses that showed higher overall trait-based population differentiation (Table 2 and Table 3) and higher F_ST_ in *E. pauciflora* than *E. ovata*, *E. ovata* showed stronger relationships between population trait means and climate variables than *E. pauciflora* (8 versus 4 significant models for the 10 performance and leaf traits, Table 6). 

**Figure 2:**
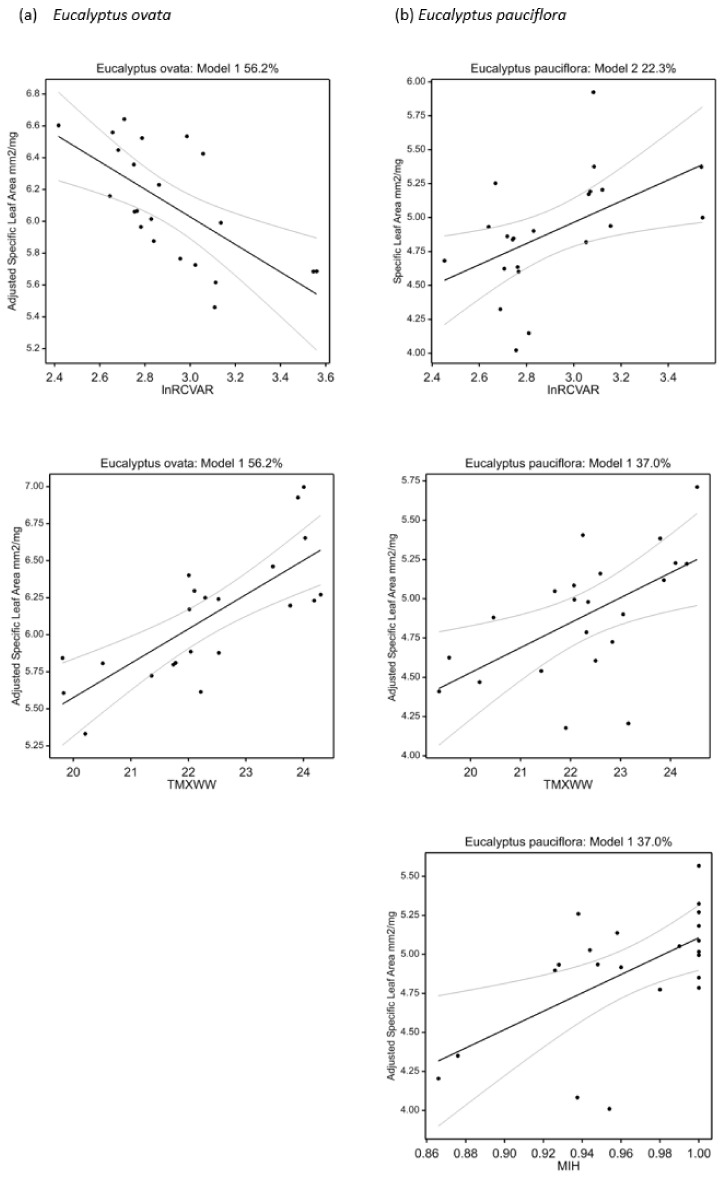
Partial dependency plots for selected regression models for specific leaf area: (**a**) *E. ovata*, and (**b**) *E. pauciflora* (see Table 6). The percentage of the total variance explained (R^2^) in the response variable is provided for each full model, and the relationships of specific leaf area with each of the climate predictor variables included in each model are shown (adjusted for other predictors modelled where appropriate). The overall models and their regression parameters for the climate predictors were statistically significant at *p* < 0.05. The 95% confidence intervals are also shown.

The best regression models for *E. ovata* showed a clear pattern of greater SLA, lower leaf thickness and density, and denser and smaller stomata with increasing home-site maximum temperature (TMXWW) and, in some cases, with lower home-site precipitation seasonality (lnRCVAR) (Table 6 and Table S4, Figure 2). These trait patterns concurred closely with growth performance trends in *E. ovata*, with maximum height and stem diameter also increasing with home-site maximum temperature (TMXWW) (and mean annual precipitation, lnRANN) and declining with home-site precipitation seasonality (lnRCVAR) (Table 6, Figure S1). Other trends included increasing vein density with home-site temperature (TANN), as well as increasing stomatal density with home-site wettest quarter temperature (TWETQ) and precipitation seasonality (lnRCVAR). These patterns are largely consistent with expectations of a less conservative growth strategy [47,48,49] and concur with the discriminant analysis, apart from a nonsignificant correlation with precipitation seasonality in the discriminant analysis.

**Figure 3:**
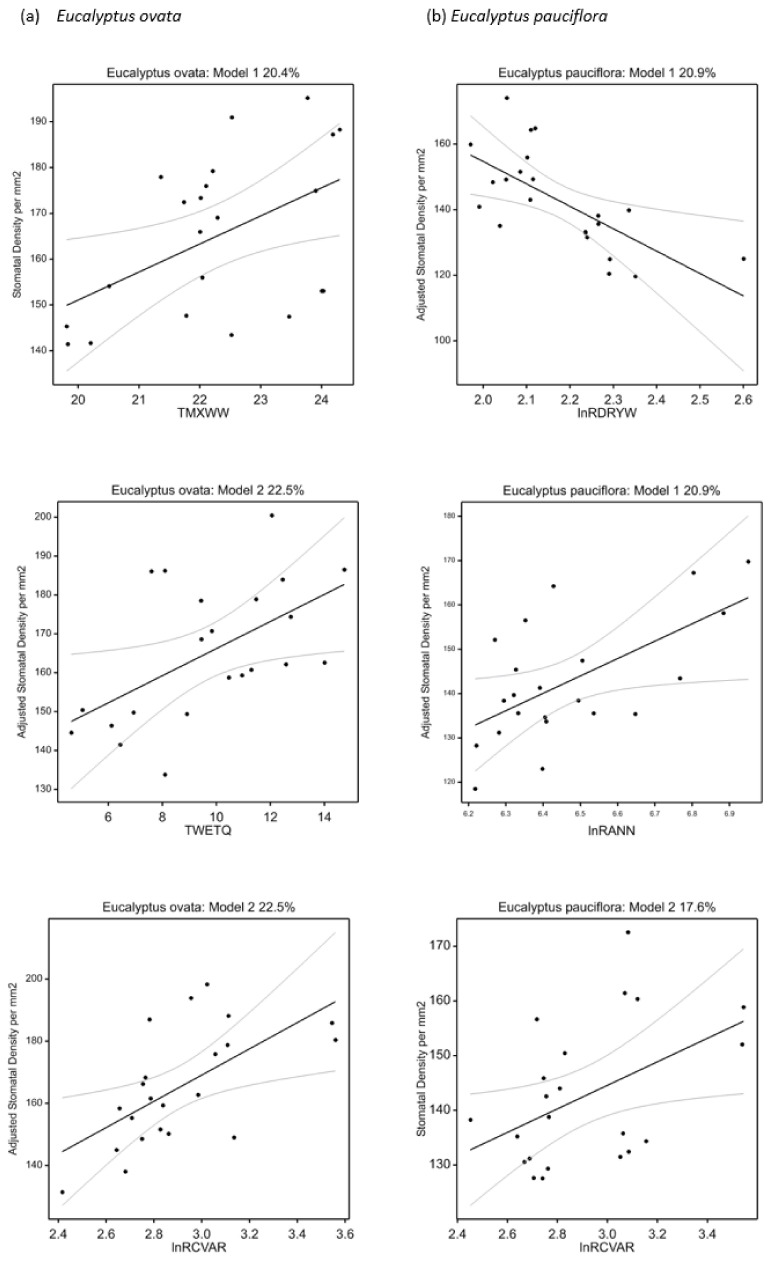
Partial dependency plots for selected regression models for stomatal density: (**a**) *E. ovata*, and (**b**) *E. pauciflora* (see Table 6). The percentage of the total variance explained (R^2^) in the response variable is provided for each full model, and the relationships of stomatal density with each of the climate predictor variables included in each model are shown (adjusted for other predictors modelled where appropriate). The overall models and their regression parameters for the climate predictors were statistically significant at *p* < 0.05. The 95% confidence intervals are also shown.

Best climate-trait regression models for *E. pauciflora* concurred with *E. ovata* models in suggesting a significant increase in SLA and lower leaf thickness with increasing home-site maximum temperature (TMXWW) (Table 6, Figure 2). This was consistent with a role for maximum temperature more generally on the *E. pauciflora* discriminant analysis, and potentially aligned with a marginally nonsignificant (*p* = 0.086) increase in height with home-site temperature (TANN) (Table 6, Figure S1). On the other hand, most other aspects of the *E. pauciflora* best regression models differed from those for *E. ovata.* This includes an increase in SLA and decrease in leaf thickness with increasing moisture index of the wettest period (MIH) not detected in *E. ovata*, and an opposing relationship with precipitation seasonality (unexpectedly, SLA increased and leaf thickness decreased with increasing lnRCVAR) compared with the trend in *E. ovata* (Table 6, Figure 2). Further, stomatal traits showed stronger relationships with precipitation-related variables in *E. pauciflora* compared with a stronger relationship with temperature variables in *E. ovata*, again consistent with the discriminant analysis (Figure 1). These included increased stomatal density with higher home-site seasonality (lnRCVAR) and precipitation (lnRANN) (countered by lower precipitation in the driest week, lnRDRYW), and higher stomatal length per unit area with home-site aridity (temperature in the driest quarter, TDRYQ) (Table 6, Figure 3).

## 3. Discussion

We hypothesised that our two co-occurring study species would demonstrate similar patterns of trait variation and trait–climate relationships owing to their shared distribution subject to the same macro-climate gradients. Our common-garden study revealed only weak support for this hypothesis, and instead revealed strongly disparate patterns of genetic-based population variation and different climate–trait associations between *E. ovata* and *E. pauciflora*. Even when statistically significant population differentiation in leaf traits was detected in both species, the spatial patterns of population variation were: (i) not correlated between species; (ii) usually associated with different facets of the variation in climate; and (iii) generally independent of the patterns of genomic differentiation. 

Such decoupling of the patterns of population variation in functional traits in these two co-occurring eucalypts contrasts with previous reports of parallel patterns of in situ population variation in co-occurring eucalypt species. These include clines in foliar leaf waxiness (glaucousness) on several Tasmanian mountains [50], as well as leaf functional and physiological trait variation along a precipitation gradient on mainland Australia [51]. There are few similarly paired common-garden studies of trees with which to compare our study. A conceptually similar study is that of Vitasse et al. [52] involving common-garden comparisons of provenances of six co-occurring species sampled along altitudinal gradients in two valleys in the Pyrenees. While many traits showed parallel altitudinal/temperature clines, importantly they did find opposing clines signalling different relationships among traits and fitness components in different species (see also [53]). Similarly, in common-garden experiments, the diversity among northern hemisphere tree species in the slopes of clines in leafing phenology with home-site altitude and latitude is evident in reviews by Salk [54] and Alberto et al. [5]. Even within the same species, common-garden trials have demonstrated differing patterns of population differentiation with respect to the same environmental gradient [55]. Further, species-level studies focusing on measurements in situ or in glasshouse studies (rather than in common gardens) have similarly suggested that mechanisms for adaptation to climate can differ between congeneric species along the same climate gradient or in response to the same stressors [56,57,58]. 

### 3.1. Potential Drivers of Disparate Patterns between Species

Historical, genetic, or ecological factors may explain our observed decoupling of patterns of population variation within co-occurring species. First, it is possible that the population differentiation observed largely reflects neutral processes through genetic drift in small populations or different historical migration patterns. However, as the two species are widespread throughout Tasmania and are likely to have been subject to similar distributional changes during and following the last Pleistocene glaciation of the island [59], we expected a positive association of patterns of population variation. Further, the Tasmanian populations of both species exhibit very low differentiation in SNP and microsatellite markers [60] (reports of species-wide F_ST_ values include: microsatellites—a mean value for Australian tree species of 0.13 ± 0.01 [61]; SNPs—0.04 and 0.06 for *E. marginata* and *E. globulus*, respectively [62,63]). On average, these marker types are generally expected to reflect neutral variation [60], suggesting that neutral population processes are unlikely to have shaped the contemporary patterns of population differentiation of functional traits. Our sampling design did not allow direct estimation of narrow-sense heritabilities to link our estimates of inter-population variability in functional traits based on the intra-class correlation coefficients (Table 2) to the Q_ST_ measure of population divergence, that is comparable to the genetic differentiation at putative neutral molecular markers measured by the F_ST_ [60]. However, following Leinonen et al. [64], if we conservatively assume heritabilities of 0.5 and 1, the estimated Q_ST_ for our traits showing statistical significance would be two to nine times greater than the 95% upper confidence limit of the F_ST_ estimate (based on the microsatellite estimates for *E. pauciflora* this is 0.039—see results). Such Q_ST_ > F_ST_ differences, signal adaptive population differentiation due to directional selection [60], and have been found in other studies relating population variation in tree species with climate (reviewed in [5]; see also [10,65]). Our conclusion that the infraspecific population variation we observed in our study species reflects divergent selection is further supported by the lack of statistical significance we detected in most cases for a correlation between the pairwise matrices of quantitative and molecular population divergence within species (Table 5), as also found in other studies [65,66]. 

Second, the two species may differ in their patterns of climate adaptation due to different patterns of correlated evolutionary changes among the focal traits during species divergence (i.e., evolutionary integration [21,22,67]). In addition, for a given species, genetic constraints associated with genetic architecture within populations may have influenced the course of phenotypic evolution and adaptive diversification of the conspecific populations [68,69,70]. It is also possible that, within a species, differences among populations in natural selection may have shaped the genetic (co)variance patterns within populations over evolutionary time, thus leading to an alignment between genetic architecture and population trajectories [71,72,73]. For example, for a set of seedling traits, different directional selection gradient estimates arising from imposed acute drought stress have been reported for glasshouse-grown mesic and arid populations of *E. pauciflora* [74]. Similarly, at the species level, two intermixed oak species in the same stand have been shown to exhibit significant differences in direction of the selection response and potential for adaptive evolution [75]. *Eucalyptus ovata* and *E. pauciflora* differ significantly in virtually all leaf traits studied, which may also be indicative of the possibility of different developmental/functional integration of leaf traits at the among- and/or within-population levels for climate adaptation [21]. 

Third, it is possible that species differences in phenotypic plasticity contribute to the observed discordance in patterns of populations variation [6,76]. We compared the species based on their patterns of genetic-based population differentiation in a single common garden. However, genotype-by-environment (GxE) interactions in the measured functional traits (i.e., variation in phenotypic plasticity among populations, e.g., [49]) may have differentially masked genetic-based variation in one-or-other species for a trait. This is possible as GxE interactions at the species and population levels have been reported in eucalypts and other tree species for many of the traits we have studied ([77,78,79,80], but see [81]). The inter-trait correlations can also vary with the test environment [51]. Thus, we cannot dismiss the possibility that the observed species differences may reflect alternative climate adaptation/acclimation strategies, for example, with *E. ovata* exploiting plasticity (rather than genetic adaptation) to a larger extent than *E. pauciflora*. 

Fourth, species differences in climate adaptation strategy may also occur at the mechanism/trait level [58,67,82,83]. For example, as predicted by our second hypothesis [33,35], weak and inconsistent correlations among individual hydraulic and economic traits, as well as different relationships with climate variables, confirmed that patterns of trait responses reflected different axes of variation for leaf hydraulic versus economic traits. Further, the two species showed differing degrees of population variation in leaf economic versus hydraulic traits, suggesting differential species-level exploitation of these trait dimensions. Beyond leaf and hydraulic traits, there are also many other mechanisms that affect the ability of plants to persist under stress or environmental change [36,37]. Of note are the vegetative recovery mechanisms that eucalypts possess to recover from crown damage or death including lignotubers and epicormic buds [84,85]. Indeed, significant climate-associated population variation in seedling lignotuber development was reported in both studied species and related to climate variables such as maximum temperature that were prominent in our models [23,86,87]. 

A final explanation for the differences in the amount and pattern of population differentiation is that the different habitats occupied by the two species may differentially modulate climate-driven selection [88,89]. Variation in soil water holding capacity is an obvious example [90], but other climate–soil interactions may be involved. For example, in an early study of *E. pauciflora*, it was suggested that differences in air and soil temperature may change the interplay between leaf transpiration and water uptake by the roots [91]. While *E. ovata* showed less overall population variation in the studied leaf traits than *E. pauciflora*, the trait variation in *E. ovata* was typically more strongly related to climate. Given our conclusion that much of the trait variation is likely to reflect adaptive divergence, *E. pauciflora* may be more strongly differentiated by nonclimate factors varying across the better-drained soils it occupies, including abiotic factors such as soil depth or type ([92]; for an example of drought susceptibility, see also [90]). In contrast, the seasonally water-logged habitat of *E. ovata* may be more homogeneous across populations but allow the impact of climate variation on populations to be better expressed. Such modulation could explain: (i) the lower population differentiation across the range of *E. ovata* compared with *E. pauciflora*; and (ii) why different traits and facets of climate contributed differentially to population divergence in each species. The latter is potentially reflected in the differing relationships of SLA and leaf thickness with climate in the two species. While both species produced higher SLA and thinner leaves with warmer home-site maximum temperatures, consistent with observed better growth performance in plants from warmer environments, this pattern was superimposed on contrasting trait associations with moisture index and precipitation seasonality that could be modulated by soil factors. Biotic habitat factors such as differences in the associated enemy and plant community may also alter the effects of climate-driven selection on a focal species [93,94]. In Tasmanian eucalypts, for example, the potential for co-occurring rainforest species to affect clines in foliar glaucousness was noted early [50], and a recent study showed that drought damage in two focal eucalypt species is differentially influenced by neighbouring tree species [95].

### 3.2. Associations between Climate and Trait Variation

Given that directional selection is likely to contribute to the observed patterns in population divergence in leaf traits, a key question addressed by our third hypothesis is whether trait patterns in *E. ovata* and *E. pauciflora* reflected expectations for adaptation to climate. We found this is possible for a range of traits, especially for *E. ovata* where up to 58% of the total trait variation among population means could be explained by climate variables. At the same time, lower percentages of the total trait variation were explained by climate in *E. pauciflora*, suggesting that nonclimate factors may also be important.

Traits were often associated with multiple climate variables within each species. Taken together, these suggested several patterns of climate adaptation in the study landscape. The integrated signal revealed in *E. ovata* indicated that populations that originate from home-sites with higher maximum temperatures (and less seasonally variable or more precipitation) tend to be faster growing and to have characteristics such as higher SLA, less dense and thinner leaves, smaller and more stomata, and greater vein density. All of these characteristics are consistent with a less conservative, faster growth strategy [27,29,33]. Climate-related trends in *E. pauciflora* were weaker and suggested more prominent roles of precipitation-related variables, especially for leaf hydraulic traits.

While other recent large-scale, common-garden studies of population variation in forest tree species have shown intraspecific relationships between climate and hydraulic leaf traits [96,97], few common garden studies have investigated intraspecific variation in leaf hydraulic traits in eucalypt species with which to compare our results. However, population studies of associated physiological traits such as photosynthetic rate, stomatal conductance and surrogates of water use efficiency have reported varying degrees of genetic variation in these traits [35,43,98,99]. 

Stomata size is frequently linked with whole plant function, for example, smaller size is associated with greater capacity for CO_2_ absorption and greater speed of response to environmental signals [48]. In our study, trends in stomata size (i.e., guard cell length) were more prominent in *E. ovata*, where decreasing size with home-site maximum temperature and precipitation seasonality is consistent with faster growth rates and potential for more rapid control of water loss in extreme temperatures or drought. Population differences involving lower drought susceptibility with smaller stomata have been reported in other trees [96,100], and in eucalypts, stomatal size has been observed to decrease with decreasing driest quarter precipitation at the population home-site climate [78]. 

Vein density, representing investment in water transport and ultimately maximum photosynthetic rate [33,34,101], similarly increased with home-site (mean) temperature in *E. ovata*. This is consistent with more general expectations that environmental factors that increase transpiration of plants or decrease water availability increase the leaf venation density [47]; although a previous study of wild eucalypt populations found vein densities increased with increasing home-site aridity rather than temperature as in our study [102]. No trends were detectable in *E. pauciflora*, more consistent with the absence of a vein density-climate association reported among species of the Australian Proteaceae [39].

Stomatal density was the main leaf hydraulic trait associated with climate-related population differentiation in both of our study species. In *E. ovata* it was related to home-site temperature, consistent with reports for other tree [97] and shrub [103] species. Population mean stomatal density was positively correlated with stomatal length per unit leaf area and vein density, consistent with coordinated water transport, with both traits affecting the maximum rates of photosynthesis and water loss [104,105]. However, while the variation in stomatal density may reflect adaptation for faster growth in warmer home-sites, it could also reflect local adaptation for ‘heat avoidance’ through evaporative cooling [26,103,106]. While Tasmania has a cool-temperate, oceanic climate, this possibility cannot be dismissed as maximum temperatures in summer can average nearly 30 °C in inland regions [107] and population variation in all stomatal traits in *E. ovata* was associated with home-site maximum temperature (TMXWW). Such adaptation may explain the stronger relationships of hydraulic traits with temperature in *E. ovata* but not in co-occurring *E. pauciflora*, as evaporative cooling may be advantageous in some of the seasonally waterlogged habitats in which *E. ovata* grows, but deleterious in more water-limited *E. pauciflora* habitats [108]. Indeed, stomatal density was more strongly associated with moisture-related climate variables in co-occurring *E. pauciflora*, with stomatal density increasing with increasing home-site precipitation seasonality and aridity (i.e., lower precipitation of the driest week, RDRYW), which could reflect an adaptation for fast growth when sufficient soil water is seasonally available ([109]; see below). 

The leaf economic traits generally contributed more to population-level differentiation and climate adaptation in *E. ovata* and *E. pauciflora* than did the leaf hydraulic traits (e.g., comparisons of adjusted R^2^ values Table 6), as evident in other forest tree studies (e.g., [65]). In contrast to the hydraulic traits, there are many studies reporting significant population differentiation and climate-trait associations involving the leaf economic traits studied. A common trend in evergreen species is for leaf area and SLA to decrease and leaf thickness to increase with decreasing home-site precipitation [10,110,111,112], with more sclerophyllous leaves (low SLA) thought to have more conservative water use [113]. In eucalypts, this precipitation-trait association has been reported at the population level in a number of common-garden studies [77,79,98]. 

On the other hand, in the two evergreen species we studied, SLA increased and leaf thickness decreased with increasing home-site maximum temperature (and potentially growth rates, Table 4). This is a less commonly reported trend but has been reported for maximum temperature in *E. microcarpa* [114] and for minimum temperature in an evergreen *Pistacia* species [88]. In the landscape studied, the temperature trend was superimposed on different associations with precipitation seasonality (*E. ovata*) and moisture index of the wettest quarter (*E. pauciflora*), which could be interpreted as populations from hotter home-sites with seasonally uniform precipitation (*E. ovata*) or high soil water availability (*E. pauciflora*) evolving more towards a faster growth strategy. Nevertheless, when precipitation seasonality was modelled alone in *E. pauciflora*, it showed opposite trends to *E. ovata* with respect to precipitation seasonality, with SLA increasing and leaf thickness decreasing with increasing precipitation seasonality. This suggests that the adaptive strategy in populations of *E. pauciflora* from home-sites with more seasonal precipitation allows for fast resource acquisition and growth rates when sufficient soil water is available, analogous to that reported for seasonally arid populations of a neotropical oak [36,109]. Such recovery from seasonal water stress may involve repair and recovery of hydraulic function, which may be rapid in *E. pauciflora* [115]. 

In the cool-temperate climate in which our study species grow, frost is also a selective agent [107]. Frost will impact the other extreme of the temperature gradient and could reinforce the temperature associations involving leaf economic traits. Average winter temperatures are highly correlated with the temperature variables included in our analysis (TMXWW and TANN; r > 0.8) and there is evidence in *E. pauciflora* that thicker leaves are less susceptible to internal freezing [116], consistent with our observations of thicker leaves in both study species at lower (maximum) temperatures. Further, leaf density also increased with decreasing home-site maximum temperature in *E. ovata*. Increasing leaf density generally reflects smaller cells with thicker walls, and relatively less intracellular air space [110,117], which could thus represent an additional adaptive pathway to cold or frost tolerance, as also found for *E. tricarpa* [79].

### 3.3. Conclusions and Implications

We showed that patterns of functional trait differentiation of populations within tree species that co-occur across the same geographic and climatic landscape are uncorrelated. A component of the differentiation in functional traits is likely to have arisen through climate-driven directional selection, consistent with a range of other studies in eucalypts [43,118] and other species [5,7,11,12,119]. On the other hand, the lack of correlation in patterns of population variation in the two species when they co-occurred, and the associated differences in climate-trait associations, suggest that the trajectories of climate-driven local adaptation are different. This may be due to historical, genetic, and/or ecological factors. Indeed, with the two species occupying different habitats and markedly different in growth and leaf economic traits, it is likely that climate stressors impact species differently even when they co-occur in the same landscape. 

Importantly, while the level of population differentiation and climate-trait associations detected in our study signal historic climate adaptation, at this early stage in the life cycle of our study plants, links with growth performance were only beginning to emerge. Indeed, with long-lived trees such as our study species, a key challenge remains in linking the observed variation in functional traits to long-term fitness consequences at the individual [120] and population levels. 

Finally, our results have two clear implications for climate-based provenancing strategies. First, evidence for climate-driven directional selection within our study eucalypts, consistent with findings for other plant species, supports the potential value of capturing this adaptive diversity through provenancing strategies that increase climate resilience (e.g., [4,121]). Second, the demonstrated lack of correlation in climate-trait associations between our co-occurring study species indicates that specific patterns of climate adaption are difficult to predict. We thus propose that selection of populations for climate-resilient plantings needs to focus on sampling broadly across gradients reflecting multiple facets (e.g., temperature and precipitation means, extremes, and seasonality) of projected future climates. This strategy would maximise the climate-adaptive variation captured in forestry and ecological renovation investments until further generalisations or species-specific information is available for weighting the importance of the key climate variables for provenance selection (e.g., [122]).

## 4. Materials and Methods

### 4.1. Study Species

The study species were *Eucalyptus pauciflora* subsp. *pauciflora* (hereafter, *E. pauciflora*) and *E. ovata* subsp. *ovata* (hereafter, *E. ovata*), the main tree species being used in ecological restoration projects in one of the most arid regions of Tasmania [118,123]. They are from different, reproductively isolated [124] subgenera (subgenus *Eucalyptus* and subgenus *Symphyomyrtus*, respectively) and are thus phylogenetically and genetically independent. Both species are widely distributed in and native to south-eastern Australia. *Eucalyptus pauciflora* has one of the broadest altitudinal ranges of any eucalypt (i.e., growing from sea-level to near the alpine tree line), whereas *E. ovata* is replaced by other species at higher altitudes. At mid-altitudes, these species co-occur across large components of the geographic range of their natural distributions in Tasmania, but occupy different ecological niches within the landscape [125]. *Eucalyptus ovata* occurs on seasonally poorly-drained sites and is replaced by *E. pauciflora* on well-drained sites. 

### 4.2. Source Populations

**Figure 4:**
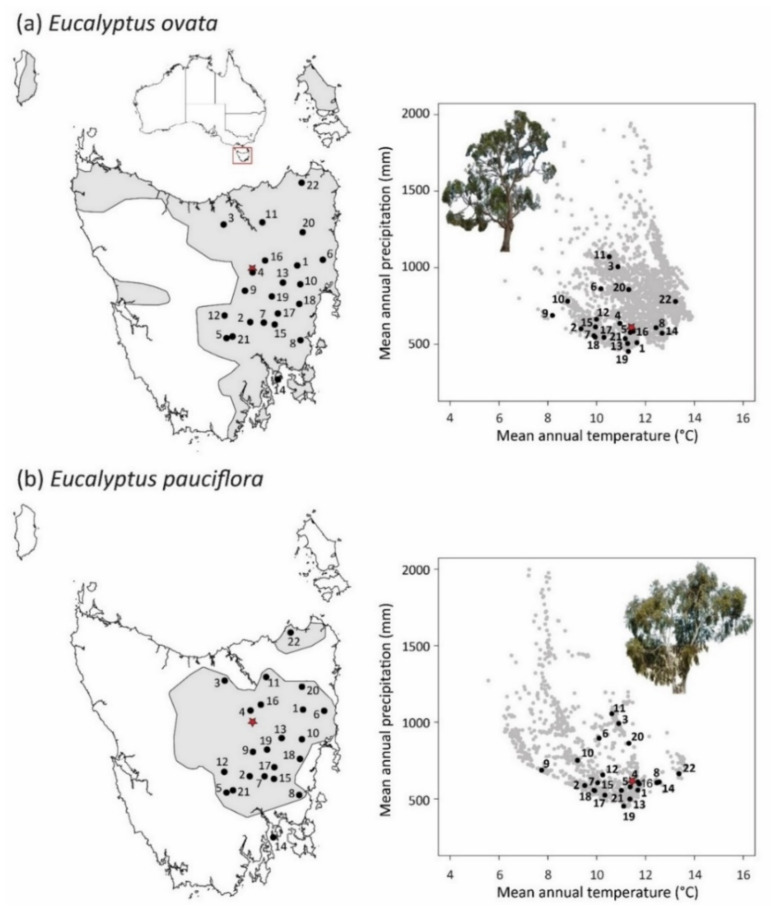
Maps of the Tasmanian geographic range and climate space for: (**a**) *E. ovata*, and (**b**) *E. pauciflora*, showing the distribution of the sampled 22 paired-localities (black points; see Table S3) and the common-garden site (red star). The climate plots show the mean annual precipitation (RANN) and temperature (TANN) of the populations included in this study, superimposed on distributional records of each species, obtained from the Natural Values Atlas (accessed August 2015). (Images: P.A. Harrison).

The source populations studied were distributed across the overlapping climatic and geographic ranges of *E. ovata* and *E. pauciflora* within Tasmania; this region of overlap is centred in the Tasmanian Midlands. Within this region, we sampled 22 locations where *E. ovata* and *E. pauciflora* occurred in close geographic proximity (Figure 4, Table S3). This pairing of populations ensured that, while the species occupied different local habitats, the macro-climatic gradients experienced by the populations of each species were effectively the same (hereafter, ‘common climate landscape’). The 22 sampled populations of *E. ovata* ranged in altitude from 9–746 m (Table S1) and encompassed virtually the full altitudinal range reported for *E. ovata* in Tasmania [125]. They were spread across the full range of mean annual temperature (TANN) experienced by wild populations of *E. ovata* in Tasmania, and covered the annual precipitation range (RANN) experienced by much of the species distribution, except for high precipitation outliers (Figure 4). The sampled populations of *E. pauciflora* extended over a similar altitudinal range (16–824 m, Table S1), but as *E. pauciflora* extends to higher altitudes than *E. ovata* [125,126], the higher altitudinal component of the *E. pauciflora* distribution, representing the lower temperature extremity, was not represented in our paired study. Further, as our study species also occur in mainland Australia, the upper extremities of mean annual temperature in Tasmania (c. 14 °C) did not represent the extremities of their broader range in Australia (c. 17.5 °C). Hence our study did not aim to investigate the significance of adaptation in range-edge populations, which have in some studies been found to be the most adaptively differentiated [80,127].

### 4.3. Experimental Common Garden

The provenance trials of the two eucalypt species (*E. ovata* and *E. pauciflora*) studied were part of a set of multi-purpose common-garden trials established at Connorville, Tasmania (41.828° S, 147.138° E, altitude 185 m) [123]. The common garden represented a climate at the drier, mid-temperature range of the sampled provenances (Figure 4). The seedlings used to establish the common garden were grown for 9–11 months (*E. ovata*) and 16–17 months (*E. pauciflora*) in outdoor nursery conditions, then planted at Connorville on 6–8 August 2014. The trials were established on an ex-pasture site, likely to have been dominated originally by a mix of *E. viminalis* and *E. amygdalina* with the occasional *E. ovata* and *E. pauciflora*. The area was surrounded with deer proof fencing to exclude large vertebrate herbivores. Within this area, each of the field trials was established with eight blocks, with the blocks of each species spatially paired across the planting area. Each block contained up to 360 families derived from single-tree open-pollinated seed collections from native populations across their shared geographic range in Tasmania [123]. Families were randomly allocated as single-tree plots within each block of a given trial. Our study was based on a subsample of each of the focal 22 populations of either species. Each subsample per population included one plant from each of five blocks (110 plants per species, with the five individuals sampled per population representing five unique families).

### 4.4. Trait Measurements

Sampling at the common garden was undertaken when plants were two years old (June 2016). We quantified a suite of whole plant performance measures, leaf economic traits and leaf hydraulic traits for each sampled individual, as described below.

#### 4.4.1. Growth Performance Measures

Plant height and diameter were measured approximately two years after planting, on 1–2 June 2016. We measured the diameter of each stem at 0.5 m above ground level, and the height of the tallest stem using height poles. Total stem cross-sectional area was calculated from diameter data. Many of the trees had multiple stems from just above ground level, and in these trees the cross-sectional area of each stem was calculated, and following Davidson et al. [128] a single-stem diameter equivalent was derived from the sum of these cross-sectional areas.

#### 4.4.2. Leaf Traits

For each individual, 13 fully expanded leaves excluding petioles were collected from three branches distributed around the mid-outer, sun-exposed part of the canopy [79,129]. Once collected, three leaves per individual were immediately placed into the fridge at 4 °C in double zip lock bags for analyses of stomatal and venation traits, whilst the remaining 10 leaves were stored in the fridge for the measurements of leaf economic traits.

The measured leaf economic traits, including leaf thickness, leaf area, leaf dry mass, specific leaf area (SLA), and leaf tissue density, were calculated from the dry mass, thickness, and area values. Surface area without petiole was estimated by scanning the leaves using a portable scanner (Brother, MCF-J65200W, Japan); the area of each leaf (cm^2^) was determined using Matlab (MathWorks, Natick, MA, USA). Leaf thickness (mm) of fresh leaves was measured using digital callipers (Mitutoyo, Japan), avoiding the influence of leaf major veins. Sampled leaves were then oven-dried at 70 °C for at least 48 h to a constant mass. The mass of each dried leaf sample was estimated with a precision of 0.1 mg. Leaf data were averaged across the 10 sampled leaves to give a single value of each trait for each tree.

Leaf hydraulic traits, including stomatal density and leaf venation, were measured on three leaves per individual (the same leaves were used for both). Samples for stomatal observations were made using the nail polish impression method as described by Franks et al. [130]. Leaves were found to be amphistomatal and epidermal impressions were taken from both sides of the leaves. Stomatal density (i.e., number of stomata per unit epidermal area in mm^2^) was calculated for each leaf as the mean of six fields of view (three on each side) at 270× total magnification using a light microscope and USB eyepiece camera (Olympus, Tokyo, Japan; Dino-Eye AM423, Hsinchu City, Taiwan). Stomatal pore length (µ) (between the guard cells) was measured as the mean of 20 stomatal complexes (guard cell pairs) for each leaf (10 on each side) at 1000× magnification using the same light microscope. Total stomatal length (µ mm^−2^) was calculated from stomatal density and length, as a proxy for stomatal conductance of water vapour.

Paradermal sections for leaf venation measurement were prepared according to the general protocols described by Brodribb et al. [131]. First, the adaxial epidermis and palisade mesophyll were removed using a sharp razor blade to expose the minor veins. Sections were then placed in bleach (50 g L^−1^ sodium hypochlorite and 13 g L^−1^ sodium hydroxide) for several hours until clear. After clearing, sections were carefully rinsed to remove bleach and stained in 1% toluidine blue for 30 s to colour the lignin-rich veins. Finally, sections were photographed with a (Dino-Eye AM423, Hsinchu City, Taiwan) USB camera mounted on a (Olympus, Tokyo, Japan) microscope at 270× total magnification. Vein density was measured in five fields of view as the total length of leaf venation per leaf area (mm mm^−2^) using ImageJ (NIH Image, Bethesda, MD, USA) by manually drawing and counting all veins in the field.

### 4.5. Molecular Methods

Leaf tissue was collected from up to 10 wild mothers per population; these were mothers of the progeny represented in the trial. The leaf tissue was snap frozen in liquid nitrogen, then stored at −80 °C until required. High quality genomic DNA was extracted from frozen samples using a modified CTAB protocol [132,133]. As leaf samples were not available for one of the *E. pauciflora* populations studied (population 14 in Figure 4), the genomics-based analyses were restricted to the 21 paired populations common to both species. DNA from *E. pauciflora* was genotyped using DArTSeq (Diversity Arrays Technology combined with next-generation sequencing technology; [134]). DNA from *E. ovata* was genotyped using the Thermo-Fisher Axiom^TM^ *Eucalyptus* Genotyping Array (Axiom Euc72K chip, https://www.thermofisher.com/order/catalog/product/551134; accessed on 10 April 2022). The single nucleotide polymorphism (SNP) genotype data from both sources were filtered to a species-wide minor allele frequency of 0.05 and <5% missing data, following Ahrens et al. [135]. This filtering yielded 3603 (*E. pauciflora*) and 22,708 (*E. ovata*) SNP markers. An additional dataset of 10 putatively neutral nuclear microsatellites (SSR) was available for the same individuals of *E. pauciflora* [45]. The SNP and SSR data were derived from up to 10 parental trees per population, 5 of which were the parents of the progeny sampled in the present study. Using population-level frequency data, a wrapper function in R was written to estimate Weir and Cockerham [136] species-level F_ST_ and population-level pairwise F_ST_ values using the ‘wc’ function of the *hierfstat* package [8]. This was also used to estimate 95% confidence intervals, which were obtained using 1000 bootstrap resamples of the data.

### 4.6. Climate Data

The 35 BIOCLIM variables representing temperature, precipitation, radiation, and moisture over the period 1976–2005 were obtained for each sampled mother tree within a population using each mother’s geographic location (GPS coordinates) and elevation (estimated from a 9 sec digital terrain model), and the program ANUCLIM v. 6.1 [137], setting the period to a week and assuming clay soils (Table S5). Four additional climate variables were extracted from spatial layers accessible through the Atlas of Living Australia (ALA; Table S5) using the *ALA4R* package in R [138]. Climate variables were averaged across the five trees sampled per population to provide population-level estimates. 

The distributions and ranges of data for each climate variable were checked using histograms, leading to natural log transformation of precipitation variables to ameliorate left skewness, and exclusion of isothermality (TISO) due to its minimal range (Table S5). We used an iterative process to reduce the number of remaining climate variables to a subset that represented the variation in the climate data. This was achieved by first dividing the climate variables into temperature-related, precipitation-related, and other-climate groups. Within each group, we examined pairwise correlations, and identified sets of variables with absolute correlations |r| > 0.80. From these sets, we selected a single variable for retention, favouring commonly used variables with tangible biological importance. Correlations were then examined among all variables remaining in the 3 selected sets, and further reductions were made by the same method, resulting in 10 climate variables selected for further analysis (Table 7).

### 4.7. Data Analysis

#### 4.7.1. Species Differences and Within-Species Population Variance in Growth Performance and Leaf Traits

The analysis of the leaf traits was based on means of the trait measurements per individual, whereas the tree observations for the growth performance traits were used. For each focal trait, a linear mixed model was fitted to the combined data across species. The fixed part of the linear mixed model included terms for species and blocks within species, whereas the random part comprised terms for population effects within species and residuals. The mixed model assumed population effects within a species to be random draws from a normal distribution, and independent of the residual terms. The random population effects and residuals were modelled by considering a heterogeneous variance structure at the species level (i.e., a heterogeneous diagonal structure specified for population and residual variances, with separate estimation by species; e.g., [139]). The population and residual variances were estimated by restricted maximum likelihood [140], and fixed-effect parameters were estimated by generalized least squares following variance component estimation. The model-based sampling (co)variance matrix of the fixed-effect parameter estimates was corrected as described by Kenward and Roger [141]. This provided standard errors for the fixed-effect parameter estimates that account for the potential finite-sample bias and uncertainty in the estimation of the variance components [141]. Based on the corrected sampling (co)variance matrix, the Kenward and Roger [141,142] approximation to compute denominator degrees of freedom was applied to improve statistical inference about the fixed-effect model terms using *F*-tests.

The distributions of the conditional residuals for the studied traits conformed well to the normal distribution, as indicated by the visual inspection of histograms and normal quantile plots. When plotted against the fitted values, studentized deleted residuals from the examined models did not reveal residual heteroskedasticity. For a given trait, statistical inference about the species effect was based on an *F*-test. The species least-squares means for the traits and their standard errors were calculated from a linear function involving the estimated fixed-effect parameters or their (corrected) sampling (co)variance matrix, respectively. Homogeneity of the population variance estimates was tested via a two-sided likelihood ratio test, which compared the full model allowing for variance heterogeneity with a reduced model where the population variances were constrained to be the same across the two species [139]. In analyses pursued separately for each species, a one-sided likelihood ratio test [143] was applied to assess whether the estimated population variance for a trait was significantly greater than zero. The intra-class correlation coefficient was calculated as the proportion of the total variance (population plus residual variance estimates) attributable to the population variance, and thus was used to measure the relative importance of the population variance for each trait. The analyses were undertaken by using the procedure GLIMMIX of SAS/STAT.

#### 4.7.2. Correlation of Population Patterns between Species

To compare the extent to which individual traits and climate variables covaried among populations within each species, Pearson’s correlation coefficients were calculated among population means for each pair-wise combination (22 populations per species) of variables for each species, using the procedure CORR of SAS/STAT.

Using the individual-level data for the six primary leaf traits (leaf area, thickness and density, stomatal density and length, vein density), comparison of population patterns was extended to the multivariate level. For each species, we undertook multivariate analyses of variance (MANOVA) and linear discriminant analysis that included populations as a classification predictor variable. These analyses also included the calculation of matrices of pair-wise generalised distances among populations. These analyses were undertaken with the SAS procedure PROC DISCRIM and for each species, separate analyses were undertaken for the leaf economic and hydraulic traits, and for these traits combined. 

Two tests were used to compare the patterns of population variation between species (with populations of the different species linked by their paired location). First, a Procrustes rotation was used to test the similarity of the ordination space defined by the first two discriminant axes derived from species-specific linear discriminant analyses. This comparison of ordination spaces was undertaken using the ‘procrustes’ function in the ‘vegan’ package of R, scaling both configurations to unit variance and selecting for a symmetric Procrustes statistic. The statistical significance of the Procrustes rotations was determined by permutation tests, using the ‘protest’ function based on 9999 random permutations [46]. 

Second, Mantel tests were used to estimate the significance of the correlation between the trait-based matrices of pair-wise generalised distances among populations within each species, with the co-located populations of the different species linked according to their location of origin. These correlations were estimated with the ‘mantel’ function of ‘vegan’ and their statistical significance tested using 9999 permutations of the data [46]. The Mantel tests were also used to compare molecular-based matrices of pairwise F_ST_ divergence among populations of the two species, as well as the F_ST_ versus generalised distance matrices of the same species. 

#### 4.7.3. Relationships between Traits and Climate

To compare specific trait/climate associations at the univariate (trait) level, for each species we used multiple linear regression analysis to model the population mean for each trait against climate variables, using the ‘All Subsets’ procedure of Genstat Version 20.0. Quadratic terms for the climate covariates were not included based on exploratory graphical observation. The best regression model for each response variable (a growth performance measure or a functional trait) and species was determined by generating all potential models with the 10 climate variables as predictors (capped at three variables per candidate model owing to limited sample size), and then selecting the model(s) with the highest adjusted R^2^ and/or lowest Bayesian information criterion (typically these were the same). We selected only from the set of models that were statistically significant overall, and in which all climate variables included had statistically significant partial regression coefficients (*p* < 0.05).

To understand the climate variables and traits most closely aligned with patterns of multivariate differentiation among populations of each species, climate and trait vectors were projected into the abovementioned discriminant space defined by the first two significant discriminant axes derived for each species, using the ‘envfit’ function of vegan in R [46]. The statistical significance of each fitted vector was tested using 9999 permutations of the data.

## Figures and Tables

**Table 1:** Estimated least-squares means (±s.e.) and results obtained from statistical testing (*F*-statistic, and associated *p*-value in parentheses) of the null hypothesis of no species effect, for the variables (performance and leaf traits) measured from 22 co-occurring populations of 2-year-old *E. ovata* and *E. pauciflora* trees growing in a common garden.

Trait	Least-Squares Means (±s.e.)		Species Effect
	*E. ovata*	*E. pauciflora*	*F*-Statistic
**Performance traits**			
Height (m)	3.11 ± 0.07	2.38 ± 0.08	44.6 (<0.001)
Stem diameter (mm)	56.5 ± 1.63	39.5 ± 1.75	50.8 (<0.001)
**Leaf economic traits**			
Leaf area (cm^2^)	20.8 ± 0.4	23.0 ± 0.8	5.70 (0.024)
Leaf thickness (mm)	0. 400± 0.004	0.506 ± 0.007	166.9 (<0.001)
Leaf density (mg/mm^3^)	0.419 ± 0.004	0.415 ± 0.004	0.6 (0.456)
SLA (mm^2^/mg)	6.11 ± 0.09	4.90 ± 0.09	88.2 (<0.001)
**Leaf hydraulic traits**			
Stomata density (N/mm^2^)	165 ± 4	143 ± 3	23.4 (<0.001)
Stomata length (μ)	30.2 ± 0.3	35.5 ± 0.4	130.6 (<0.001)
Stomata length per area (μ/mm^2^)	4858 ± 101	5002 ± 89	0.1 (0.748)
Vein density (mm/mm^2^)	13.6 ± 0.2	10.7 ± 0.2	89.9 (<0.001)

**Table 2:** Population intra-class correlations, homogeneity of population variances across species, and the Pearson’s correlations of population means between species for the variables (performance and leaf traits) measured from 22 co-occurring populations of *E. ovata* and *E. pauciflora*. The *p*-values associated with tests of whether population variances or population–mean correlations differed significantly from zero, and whether species differed in population variances, are given in parentheses. (Bolded probability values represent statistically significant results at the 5% nominal level even after the Bonferroni adjustment was applied within each species).

Trait	Intra-Class Correlation		Homogeneity of Population Variances *χ*^2^ Values	Pearson’s Correlation of Population Means between Species ^a^
	*E. ovata*	*E. pauciflora*		
**Performance traits**				
Height (m)	0.00 (1.000)	0.11 (0.069)	2.2 (0.138)	0.18 (0.432)
Stem diameter (mm)	0.00 (1.000)	0.01 (0.471)	0.01 (0.942)	0.18 (0.420)
**Leaf economic traits**				
Leaf area (cm^2^)	0.00 (1.000)	0.24 (**0.001**)	8.7 (**0.003**)	0.42 (0.052)
Leaf thickness (mm)	0.05 (0.241)	0.23 (**0.003**)	4.3 (0.037)	−0.03 (0.889)
Leaf density (mg/mm^3^)	0.11 (0.075)	0.13 (0.042)	0.0 (1.000)	0.27 (0.222)
SLA (mm^2^/mg)	0.21 (**0.004**)	0.29 (<**0.001**)	0.01 (0.903)	0.12 (0.610)
**Leaf hydraulic traits**				
Stomata density (N/mm^2^)	0.22 (**0.003**)	0.08 (0.140)	1.44 (0.231)	0.11 (0.615)
Stomata length (μ)	0.00 (1.000)	0.00 (1.000)	0 (1.000)	0.01 (0.958)
Stomata length per area (μ /mm^2^)	0.25 (**0.001**)	0.19 (**0.007**)	0.3 (0.612)	0.06 (0.795)
Vein density (mm/mm^2^)	0.06 (0.217)	0.00 (1.000)	0.4 (0.545)	−0.21 (0.347)

^a^ for comparison, the average correlation among co-occurring populations of both species for the climate variables was 0.97 (range 0.95–1.00).

**Table 3:** Multivariate analysis of variance (MANOVA) for the primary leaf economic and hydraulic traits, showing the generalized variance (Ʃλ_i_) as well as Wilks lambda and its statistical significance (*p*-value).

Leaf Trait Type	*E. ovata*			*E. pauciflora*		
	Ʃλ_i_	Wilks Lambda	*p*-Value	Ʃλ_i_	Wilks Lambda	*p*-Value
Economic	0.806	0.50	0.333	1.704	0.27	<0.001
Hydraulic	1.158	0.39	0.021	0.951	0.45	0.152
Economic & hydraulic	1.590	0.20	0.081	2.423	0.11	<0.001

**Table 4:** Pearson’s correlations among the trait population-means for *E. ovata* and *E. pauciflora* (22 populations per species).

		Height	Stem Diameter	Leaf Area	Leaf Thickness	Leaf Density	SLA	Stom. Density	Stom. Length	Stom. Length per Area
**Stem diameter**	*E. ovata*	0.69 ***								
	*E. pauciflora*	0.90 ***								
**Leaf area**	*E. ovata*	−0.15	0.20							
	*E. pauciflora*	0.11	0.02							
**Leaf thickness**	*E. ovata*	−0.52 *	−0.68 ***	0.01						
	*E. pauciflora*	−0.36	−0.32	−0.02						
**Leaf density**	*E. ovata*	0.14	−0.03	0.22	0.36					
	*E. pauciflora*	0.42	0.30	−0.16	0.23					
**SLA**	*E. ovata*	0.23	0.46 *	−0.13	−0.83 ***	−0.81 ***				
	*E. pauciflora*	0.15	0.15	0.07	−0.87 ***	−0.60 ***				
**Stomatal density**	*E. ovata*	−0.11	−0.15	0.07	0.26	−0.20	−0.05			
	*E. pauciflora*	0.02	−0.11	0.07	−0.36	−0.11	0.45 *			
**Stomatal length**	*E. ovata*	−0.06	−0.30	−0.18	0.34	0.35	−0.44 *	−0.42		
	*E. pauciflora*	0.21	0.24	0.10	0.22	0.20	−0.31	−0.35		
**Stomatal length per area**	*E. ovata*	−0.12	−0.25	0.02	0.44 *	−0.07	−0.23	0.93 ***	−0.07	
	*E. pauciflora*	0.24	0.17	0.10	−0.53 **	−0.15	0.51 *	0.72 ***	0.18	
**Vein density**	*E. ovata*	−0.30	−0.15	0.47 *	0.26	0.10	−0.22	0.55 **	−0.32	0.48 *
	*E. pauciflora*	−0.07	0.08	−0.32	−0.35	−0.43 *	0.42	0.16	−0.19	0.29

* *p* < 0.05, ** *p* < 0.01, *** *p* < 0.001.

**Table 5:** Mantel’s correlations (rho = Spearman correlation) among molecular (F_ST_) and (i) quantitative (generalized distances, GD) matrices of pairwise differences and (ii) the differences among population means of specific leaf area (SLA) and stomatal length per area (StL/A) of *E. ovata* (ov) and *E. pauciflora* (pau). Results of between- and within-species matrix comparisons are provided. The among-population generalized distance matrices of each species were calculated with all six primary leaf traits (GD_all), or the three hydraulic (hyd) or economic (eco) traits. The significance probabilities (*p*) associated with the null hypotheses (H_0_) being tested for population differentiation are indicated (21 populations per species for comparisons involving F_ST_).

Matrix X	Matrix Y	Spearman		Null Hypothesis
		rho	*p*	
Between species				
ova GD_all	pau GD_all	0.02	0.428	H_0_: Differentiation in primary leaf traits is not correlated
ova GD_hyd	pau GD_hyd	0.00	0.466	H_0_: Differentiation in primary hydraulic traits is not correlated
ova GD_eco	pau GD_eco	0.01	0.459	H_0_: Differentiation in primary economic traits is not correlated
ova GD_hyd	pau GD_eco	0.02	0.418	H_0_: Differentiation in *E. ovata* hydraulic traits is not correlated with that in economic traits in *E. pauciflora* *
ova GD_eco	pau GD_hyd	0.00	0.493	H_0_: Differentiation in *E. ovata* economic traits is not correlated with that in hydraulic traits in *E. pauciflora* *
ova F_ST_	pau F_ST_	−0.08	0.651	H_0_: SNP molecular differentiation (pairwise F_ST_) is not correlated
Within *E. ovata*				
ova GD_all	ova F_ST_	0.30	0.005	H_0_: Differentiation in primary leaf traits and SNP differentiation is not correlated
ova GD_hyd	ova F_ST_	0.07	0.244	H_0_: Differentiation in primary hydraulic traits and SNP differentiation is not correlated
ova GD_eco	ova F_ST_	0.44	<0.001	H_0_: Differentiation in primary economic traits and SNP differentiation is not correlated
ova_SLA	ova F_ST_	0.34	0.002	H_0_: Differentiation in compound trait SLA and SNP differentiation is not correlated
ova_StL/A	ova F_ST_	0.04	0.335	H_0_: Differentiation in compound trait stomata length per unit area and SNP differentiation is not correlated
Within *E. pauciflora*				
pau GD_all	pau F_ST_	0.06	0.351	H_0_: Differentiation in primary leaf traits and SNP differentiation is not correlated
pau GD_hyd	pau F_ST_	0.04	0.356	H_0_: Differentiation in primary hydraulic traits and SNP differentiation is not correlated
pau GD_eco	pau F_ST_	0.09	0.289	H_0_: Differentiation in primary economic traits and SNP differentiation is not correlated
pau_SLA	pau F_ST_	0.13	0.166	H_0_: Differentiation in compound trait SLA and SNP differentiation is not correlated
pau_StL/A	pau F_ST_	−0.15	0.873	H_0_: Differentiation in compound trait stomata length per unit area and SNP differentiation is not correlated

* i.e., do not represent different adaptation strategies to the same environmental gradient.

**Table 6:** Best multiple linear regression models relating performance and leaf traits to climate variables for *E. ovata* and *E. pauciflora* (22 populations per species). Models were selected from those where a statistically significant (*p* < 0.05) overall model fit was obtained and all climate predictors included in the model were significant at the 5% nominal level, unless only marginally nonsignificant models (*p* ≤ 0.1) were available. A maximum of three predictors were permitted per candidate model compared in model selection, and alternative models are shown when the adjusted R^2^ or Bayesian information criterion (BIC) values were similar. The signs preceding each climate predictor variable refer to an increase (+) or a decrease (−) expected in the dependent variable for a unit increase in the predictor, while holding the other predictors in the model constant. Models with a statistically significant overall model fit and the lowest BIC are bolded. Estimates of the regression coefficients are provided in Table S4.

Trait	*Eucalyptus ovata*	Adj. R^2^	BIC	*p*-Value	*Eucalyptus pauciflora*	Adj. R^2^	BIC	*p*-Value
**Performance**								
Maximum height (m)	**−lnRCVAR + TMXWW + lnRANN**	38.9	30.3	0.008	+TANN	11.3	26.6	0.086
Stem diameter (mm)	**−lnRCVAR + TMXWW + lnRANN**	41.7	33.8	0.005				ns
**Leaf economic**								
Leaf area (cm^2^)				ns	+TANN	11.3	26.6	0.069
Leaf thickness (mm)	**+lnRCVAR − TMXWW**	43.2	38.7	0.002	**−MIH − TMXWW**	42.6	26.1	0.002
Leaf thickness (mm)					−lnRCVAR	34.7	26.3	0.002
Leaf density (mg mm^−3^)	**−TMXWW**	38.9	22.8	0.001				ns
Specific leaf area (mm^2^ mg^−1^)	**+TMXWW − lnRCVAR**	56.2	26.6	<0.001	**+MIH + TMXWW**	37.0	26.0	0.005
Specific leaf area (mm^2^ mg^−1^)	+TANN − lnRCVAR + TSPAN	57.9	28.1	<0.001	+lnRCVAR	22.3	27.9	0.015
**Leaf hydraulic**								
Stomatal density (mm^−2^)	**+TMXWW**	20.4	21.7	0.020	**+lnRCVAR**	17.6	21.6	0.030
Stomatal density (mm^−2^)	+lnRCVAR + TWETQ	22.5	23.6	0.034	−lnRDRYW + lnRANN	20.9	23.3	0.042
Stomatal length (µ)	**−TMXWW − lnRDRYW**	30.0	26.4	0.013	−lnRANN	12.9	19.0	0.056
Stomatal length (µ)	−TMXWW + RRL + lnRCVAR	33.8	27.7	0.015				
Stomatal length per area (µ mm^−2^)				ns	**+TDRYQ**	17.5	21.1	0.03
Vein density (mm mm^−2^)	**+TANN**	23.9	28.2	0.012				ns

ns = *p* > 0.1.

**Table 7:** Climate variables included in the analysis (See Table S5 for sources and ranges).

Variable Code	Description (Unit of Measurement)	Ecophysiological Relevance
lnRANN	Mean annual precipitation (mm) (ln X + 1)	Catch all for precipitation-related adaptations
lnRDRYW	Precipitation of driest week (mm) (ln X + 1)	Drought exposure
lnRCVAR	Precipitation seasonality (Coefficient of variation) (ln X + 1)	Exposure to extremes in water availability
TANN	Mean annual temperature (°C)	Catch all for temperature-related adaptations
TMXWW	Maximum temperature of warmest week (°C)	Extreme heat tolerance
TSPAN	Temperature annual range (°C)	Tolerance of temperature extremes
TWETQ	Mean temperature of wettest quarter (°C)	Concurrence of moisture and growing season
TDRYQ	Mean temperature of driest quarter (°C)	Aridity/drought exposure
RRL	Lowest weekly radiation (W m^−2^)	Light limitation in coolest season
MIH	Highest weekly moisture index	Soil water availability in wettest season

## Data Availability

The data reported are being used in other studies but can be requested from the corresponding author for consideration.

## Supplementary Materials

The following supporting information can be downloaded at: https://www.mdpi.com/article/10.3390/plants11141846/s1. Table S1: Estimates of species least-squares means and standard errors (s.e.) for altitude and climate variables studied; Table S2: Results of vector projections into the ordination space defined by the two discriminant axes, derived from separate discriminant analyses of the *E. ovata* and *E. pauciflora* populations; Table S3: Location details for the paired populations of *E. pauciflora* and *E. ovata* sampled from 22 localities across their over-lapping native range in Tasmania; Table S3: Location details for the paired populations of *E. pauciflora* and *E. ovata* sampled from 22 localities across their over-lapping native range in Tasmania; Table S4: Best multiple linear regression models relating performance and leaf traits to climate variables for *E. ovata* and *E. pauciflora*, showing parameter estimates; Table S5: Climate variables extracted and considered for inclusion in the climate-trait analysis; Figure S1: Partial dependency plots for selected models for plant height for: (a) *E. ovata* and (b) *E. pauciflora*.
