# Supplementary material for: Leaf Economic and Hydraulic Traits Signal Disparate Climate Adaptation Patterns in Two Co-Occurring Woodland Eucalypts
Source: Plants (Basel). 2022 Jul 14;11(14):1846. doi: 10.3390/plants11141846 (PMC9320154; doi:10.3390/plants11141846)
Supplement: Supplementary file 1 [file plants-11-01846-s001.zip › plants-1790783-supplementary.pdf]

plants

Article

Leaf economic and hydraulic traits signal disparate climate adaptation patterns in two co-occurring woodland eucalypts

Suzanne M. Prober, Brad M. Potts, Peter A. Harrison, Georg Wiehl, Tanya G. Bailey, João Costa e Silva, Meridy R. Price, Jane Speijers, Dorothy A. Steane, René E. Vaillancourt

Supplementary Material

Table S1. Estimates of species least-squares means and standard errors (s.e.) for altitude and climate variables studied, with population minimum and maximum values being given in parentheses (. The results obtained from the significance test (*F*-statistic, and associated P-value provided within parenthesis) undertaken for testing the difference between species in a given response variable (see the footnote of the table) are also given. The statistics a presented are based on the 22 paired samples from co-occurring populations of either species. Climate variable codes follow Table A5.

	Least-squares means ± s.e.		Species effect[a] *F*-statistic
	E. ovata *(min, max)*	*E. pauciflora* *(min, max)*	
Altitude	322 ± 40	319 ± 43	0.0
(m)	(9, 746)	(16, 824)	(0.969)
TANN	10.8 ± 0.3	10.8 ± 0.3	0.0
(°C)	(8.2, 13.3)	(7.7, 13.3)	(0.957)
TMXWW	22.3 ± 0.3	22.3 ± 0.3	0.0
(°C)	(19.8, 24.3)	(19.4, 24.5)	(0.980)
TDRYQ	12.9 ± 0.65	12.9 ± 0.65	0.01
(°C)	(6.3, 17.0)	(6.3, 17.1)	(0.928)
TWETQ	9.7 ± 0.6	9.6 ± 0.6	0.01
(°C)	(4.6, 14.7)	(4.2, 14.9)	(0.919)
TSPAN	20.9 ± 0.4	20.9 ± 0.4	0.0
	(17.0, 23.6)	(17.0, 23.5)	(0.945)
RANN	660 ± 36	657 ± 33	0.01 [b]
(mm)	(451, 1077)	(501, 1042)	(0.927)
RDRYW	7.9 ± 0.3	7.9 ± 0.3	0.0 [b]
(mm)	(5.8, 15.4)	(6.2, 20.7)	(0.982)
RCVAR	18.0 ± 1.3	18.1 ± 1.3	0.0 [b]
	(10.2, 34.2)	(10.6, 34.0)	(0.984)
RRL	4.85 ± 0.04	4.85 ± 0.04	0.0
($W m^{-2}$)	(4.35, 5.14)	(4.39, 5.12)	(0.971)
MIH	0.96 ± 0.01	0.96 ± 0.01	0.15
	(0.82, 1.00)	(0.87, 1.00)	(0.700)

[a] A one-way fixed-effects linear model was fitted to population-level altitude and climate data (i.e. 22 observations per species for a given response variable), using species as a classification predictor variable) and separate (heterogeneous) residual variances for the two species. The model was fitted using the procedure GLIMMIX of SAS (Version 9.4) with a Gaussian distribution of residual variances

[b] analysis based on ln (1+X) transformed data

Table S2. Results of vector projections into the ordination space defined by the two discriminant axes, derived from separate discriminant analyses of the *E. ovata* and *E. pauciflora* populations. For a given variable, the proportion of the total variance explained by a single trait projected in the two-dimensional discriminant ordination (R^2) and the significance of this fit are provided.

	E. ovata		*E. pauciflora*	
Traits	R^2	p	R^2	p
**Performance**				
Maximum height (m)	0.0970	0.3708	0.0757	0.4649
Stem diameter (mm)	0.2622	0.0576	0.0314	0.7270
**Leaf economic**				
Leaf area (cm^2)	0.0362	0.7004	0.7659	**0.0001**
Leaf Thickness (mm)	0.7354	**0.0001**	0.8628	**0.0001**
Leaf Density ($mg\ mm^{-3}$)	0.6062	**0.0003**	0.1284	0.2745
Specific Leaf Area ($mm^2\ mg^{-1}$)	0.9167	**0.0001**	0.8220	**0.0001**
**Leaf hydraulic**				
Stomatal Density (mm^{-2})	0.9601	**0.0001**	0.5105	**0.0009**
Stomatal Length (μ)	0.4160	**0.0059**	0.1156	0.3201
Stomatal Length per area ($\mu\ mm^{-2}$)	0.8985	**0.0001**	0.5817	**0.0003**
Vein Density ($mm\ mm^{-2}$)	0.5132	**0.0010**	0.5243	**0.0011**
Climate variables				
TANN	0.2942	**0.0394**	0.2610	0.0579
TMXWW	0.5609	**0.0006**	0.3036	**0.0341**
TSPAN	0.2995	**0.0330**	0.0521	0.5986
TWETQ	0.2089	0.1096	0.0748	0.4731
TDRYQ	0.0946	0.3794	0.2332	0.0799
lnRANN	0.2811	**0.0423**	0.0999	0.3699
lnRCVAR	0.1499	0.2149	0.4199	**0.0065**
lnRDRYW	0.1693	0.1755	0.0226	0.8030
RRL	0.0539	0.5959	0.1043	0.3485
MIH	0.2839	**0.0400**	0.1641	0.1782
Geographic				
Altitude	0.3734	**0.0130**	0.3234	**0.0250**
Longitude	0.0177	0.8401	0.1523	0.2035
Latitude	0.1507	0.2152	0.1630	0.1827

Table S3. Location details for the paired populations of *E. pauciflora* and *E. ovata* sampled from 22 localities across their overlapping native range in Tasmania.

site#	*E. pauciflora*	Latitude	Longitude	*E. ovata*	Latitude	Longitude
**1**	Avoca	-41.71	147.83	Avoca	-41.82	147.75
**2**	Bignells Bothwell	-42.40	147.10	Bignells Bothwell	-42.40	147.11
**3**	Brushy Lagoon	-41.41	146.75	Brushy Lagoon	-41.39	146.73
**4**	Cressy	-41.72	147.10	Connorville Dam	-41.89	147.13
**5**	Curringa	-42.57	146.77	Curringa	-42.57	146.77
**6**	Dukes Marshes	-41.72	148.13	Coals Marsh	-41.76	148.11
**7**	Ellesmere	-42.40	147.30	Ellesmere	-42.41	147.29
**8**	Gatehouse Marsh	-42.59	147.78	The Thumbs	-42.59	147.80
**9**	Interlaken	-42.15	147.14	Woods Lake	-42.08	147.03
**10**	Lake Leake	-42.02	147.82	Lake Leake	-42.01	147.80
**11**	Nunamara	-41.37	147.32	Nunamara	-41.37	147.27
**12**	Osterley	-42.35	146.74	Osterley	-42.35	146.74
**13**	Ross	-42.00	147.53	Ross	-42.00	147.56
**14**	South Arm	-43.03	147.42	South Arm	-43.00	147.48
**15**	Stonor	-42.43	147.43	Stonor	-42.43	147.44
**16**	Symmons Plains	-41.66	147.25	Epping Forest	-41.77	147.31
**17**	Tin Dish Rivulet	-42.31	147.44	Andover	-42.31	147.49
**18**	Tooms Lake	-42.22	147.79	Tooms Lake	-42.21	147.79
**19**	Tunbridge	-42.12	147.36	Tunbridge	-42.14	147.40
**20**	Tyne River	-41.47	147.82	Tyne River	-41.47	147.85
**21**	Uralla	-42.55	146.86	Uralla	-42.55	146.86
**22**	Waterhouse	-40.91	147.66	Mt Cameron	-40.94	147.82

Table S4. Best multiple linear regression models relating performance and leaf traits to climate variables for *E. ovata* and *E. pauciflora,* showing parameter estimates. All models and parameter estimates (except constants) are significant at P<0.05 or P<0.1§ (see Table 6 for further model statistics).

**Trait**	***Eucalyptus ovata***	***Eucalyptus pauciflora***
**Performance**		
Maximum height (m)	-124.2 - 29.00 lnRCVAR + 4.96 TMXWW + 23.88 lnRANN	1.13 + 0.115 TANN§
Stem diameter (mm)	-2.07 - 1.01 lnRCVAR + 0.116 TMXWW + 0.855 lnRANN	
**Leaf economic**		
Leaf area (cm^2)		9.79 + 1.22 TANN§
Leaf Thickness (mm)	0.41 + 0.043 lnRCVAR - 0.006 TMXWW	1.31 - 0.539 MIH - 0.013 TMXWW
Leaf Thickness (mm)		0.73 - 0.077 lnRCVAR
Leaf Density (mg mm^{-3})	0.60 - 0.008 TMXWW	
Specific Leaf Area ($mm^2\,mg^{-1}$)	3.46 + 0.232 TMXWW - 0.867 lnRCVAR	-4.32 + 5.89 MIH + 0.159 TMXWW
Specific Leaf Area ($mm^2\,mg^{-1}$)	3.52 + 0.161 TANN - 0.853 lnRCVAR + 0.159 TSPAN	2.62 + 0.781 lnRCVAR
**Leaf hydraulic**		
Stomatal Density (mm^{-2})	28.5 + 6.13 TMXWW	80.0 + 21.51 lnRCVAR
Stomatal Density (mm^{-2})	8.6 + 42.2 lnRCVAR + 3.49 TWETQ	37.6 - 68.3 lnRDRYW + 39.2 lnRANN
Mean Stomatal Length (μ)	53.96 - 0.660 TMXWW - 4.13 lnRDRYW	52.07 - 0.257 lnRANN§
Mean Stomatal Length (μ)	15.18 - 0.651 TMXWW + 4.53 RRL + 2.62 lnRCVAR	
Stomatal Length per area (μ mm^{-2})		3941 + 77.5 TDRYQ
Vein Density (mm mm^{-2})	8.33 + 0.486 TANN	

Table S5. Climate variables extracted and considered for inclusion in the climate-trait analysis. The data were extracted from the climate surfaces of ANUClim (v6.1) and the Atlas of Living Australia (ALA) using the latitude and longitude of each of the five trees sampled in a population and then population averages were used in subsequent analyses.

Variable code	Description (unit of measurement)	Data source	Data range
**TANN**	Annual mean temperature (°C)	ANUClim v6.1	7.7—13.3
**TMDR**	Mean diurnal temperature range (°C)	ANUClim v6.1	8.3—12.3
**TISO**	Isothermality (diurnal vs annual temperature range)	ANUClim v6.1	0.49—0.52
**TCVAR**	Temperature seasonality (Coefficient of variation)	ANUClim v6.1	0.98—1.34
**TMXWW**	Max temperature of warmest week (°C)	ANUClim v6.1	19. —24.5
**TMNCW**	Min temperature of coldest week (°C)	ANUClim v6.1	-0.9—5.2
**TSPAN**	Temperature annual range (°C)	ANUClim v6.1	17.0—23.6
**TWETQ**	Mean temperature of wettest quarter (°C)	ANUClim v6.1	4.2—14.9
**TDRYQ**	Mean temperature of driest quarter (°C)	ANUClim v6.1	6.3—17.1
**TWMQ**	Mean temperature of warmest quarter (°C)	ANUClim v6.1	12.2—17.2
**TCLQ**	Mean temperature of coldest quarter (°C)	ANUClim v6.1	3.4—9.7
**RANN**	Annual precipitation (mm)	ANUClim v6.1	451.5—
**RWETW**	Precipitation of wettest week (mm)	ANUClim v6.1	10.2—34.4
**RDRYW**	Precipitation of driest week (mm)	ANUClim v6.1	5.8—12.5
**RCVAR**	Precipitation seasonality (Coefficient of variation %)	ANUClim v6.1	10.2—34.2
**RWETQ**	Precipitation of wettest quarter (mm)	ANUClim v6.1	127.4—
**RDRYQ**	Precipitation of driest quarter (mm)	ANUClim v6.1	99.0—210.2
**RWMQ**	Precipitation of warmest quarter (mm)	ANUClim v6.1	108.3—
**RCLQ**	Precipitation of coldest quarter (mm)	ANUClim v6.1	5.9—6.9
**RRANN**	Annual mean radiation (W m^{-2})	ANUClim v6.1	13.3—14.4
**RRH**	Highest weekly radiation (W m^{-2})	ANUClim v6.1	21.6—24.3
**RRL**	Lowest weekly radiation (W m^{-2})	ANUClim v6.1	4.4—5.2
**RRCVAR**	Radiation seasonality (Coefficient of variation)	ANUClim v6.1	46.4—51.5
**RRWETQ**	Radiation of wettest quarter (W m^{-2})	ANUClim v6.1	6.0—21.9
**RRDRYQ**	Radiation of driest quarter (W m^{-2})	ANUClim v6.1	5.9—20.2
**RRWMQ**	Radiation of warmest quarter (W m^{-2})	ANUClim v6.1	19.7—22.2
**RRCLQ**	Radiation of coldest quarter (W m^{-2})	ANUClim v6.1	5.9—6.9
**MIANN**	Annual Mean Moisture Index	ANUClim v6.1	0.5—0.9
**MIH**	Highest weekly moisture index	ANUClim v6.1	0.82—1.0
**MIL**	Lowest weekly moisture index	ANUClim v6.1	0.16—0.54
**MICVAR**	Moisture index seasonality (C of V)	ANUClim v6.1	15.8—47.9
**MIMWETQ**	Mean moisture index of wettest quarter	ANUClim v6.1	0.78—1.0
**MIMDRYQ**	Mean moisture index of driest quarter	ANUClim v6.1	0.22—0.68
**MIMWMQ**	Mean moisture index of warmest quarter	ANUClim v6.1	0.226—
**MIMCLQ**	Mean moisture index of coldest quarter	ANUClim v6.1	0.72—1.0
**AIANN**	Annual aridity index	ALA	0.6—2.2
**AIMMAX**	Maximum month aridity index	ALA	0.18—0.50
**AIMMIN**	Mininimum month aridity index	ALA	1.4—5.9
**RANNSEASONALITY**	Annual precipitation seasonality	ALA	-0.8—0.06

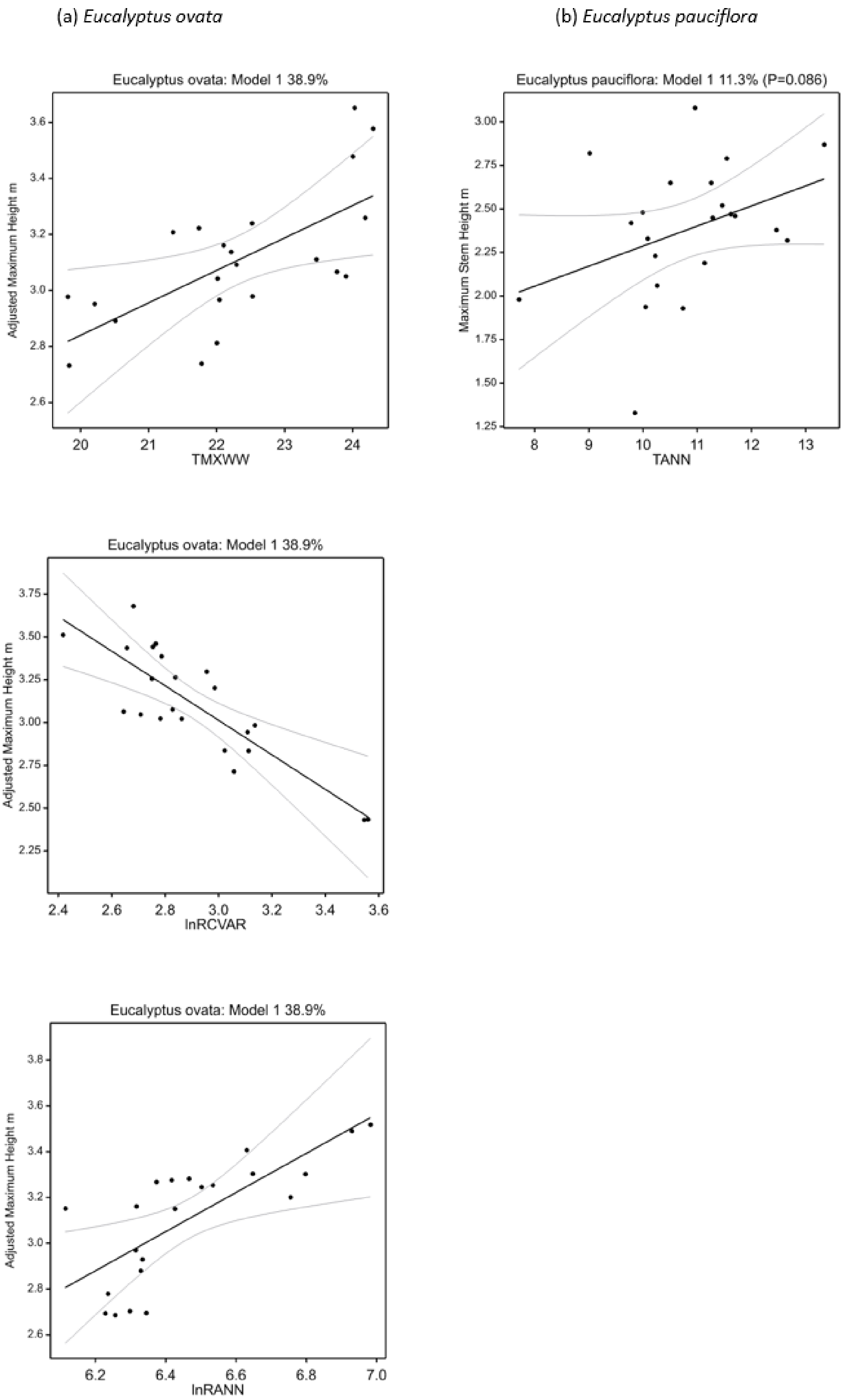

Figure S1. Partial dependency plots for selected regression models for plant height: (a) *E. ovata*, and (b) *E. pauciflora* (see Table 6). The percentage of the total variance explained (R^2) in the response variable is provided for each full model, and the relationships of height with each of the climate predictor variables included in a given model are shown (adjusted for other predictors modelled where appropriate). The overall models and their regression parameters for the climate predictors were statistically significant at $P < 0.05$. The 95% confidence intervals are also shown.
